# Potency of agarose gel‐supported lipid bilayers for electrophysiologic analysis of channel pores formed by *Bacillus thuringiensis* insecticidal proteins

**DOI:** 10.1111/febs.70070

**Published:** 2025-03-17

**Authors:** Tsubasa Okuda, Tomoya Takeuchi, Mami Asakura, Minako Hirano, Toru Ide, Tohru Hayakawa

**Affiliations:** ^1^ Graduate School of Interdisciplinary Science and Engineering in Health Systems Okayama University Japan

**Keywords:** *Bacillus thuringiensis* mosquito‐larvicidal proteins, bilayer formation at the gel/water interface, electrophysiologic analysis, lipid raft

## Abstract

Electrophysiologic analysis using artificial lipid bilayers is useful for studying the formation of pores by insecticidal proteins, especially the ion permeability of toxin pores. However, such studies are time‐consuming and require special skills, particularly regarding the construction of lipid bilayers and promoting toxin pore formation. To facilitate the analysis of toxin pore formation in the present study, we evaluated the usefulness of agarose gel‐supported lipid bilayers for electrophysiologic measurements using two structurally different mosquito‐larvicidal proteins, Mpp46Ab and Cry4Aa. The agarose gel‐supported lipid bilayers enabled the measurement of channel currents through pores made by both toxins and, notably, the lipid bilayers could be easily reconstructed even after disruption of the lipid bilayer. Using this system, measurements could be repeated at least five times using the same apparatus and toxins. We also investigated the effect of the lipid bilayer component on toxin pore formation and found that the incorporation of both cholesterol and sphingomyelin into the lipid bilayer facilitates the formation of pores by both Mpp46Ab and Cry4Aa. Both cholesterol and sphingomyelin are major components of lipid raft microdomains, suggesting that, in addition to recruiting toxin receptors, raft microdomains play a key role in membrane insertion and pore formation by insecticidal proteins.

## Graphical abstract

Agarose gel‐supported lipid bilayers constructed at the interface between the lipid solution and the recording solution are useful for measuring channel currents through different types of toxin pores. In this system, the lipid bilayers can be easily reconstructed even after the bilayer is disrupted, and measurements of toxin pores could be repeated sequentially at least five times. This is of great advantage for electrophysiologic measurements of toxin pores.

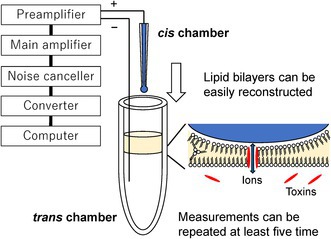

**Abbreviations**
Chol: cholesterol
GPI: glycosylphosphatidylinositol
GST: glutathione *S*‐transferase
PC: phosphatidylcholine
PFT: pore‐forming toxin
SD: standard deviation
SDS/PAGE: sodium dodecyl sulfate–polyacrylamide gel electrophoresis
SM: sphingomyelin

## Introduction

*Bacillus thuringiensis* is a Gram‐positive, spore‐forming soil bacterium that produces a crystalline protein inclusion during sporulation. Many *B. thuringiensis* strains exhibit specific insecticidal activity, and this activity is thought to be primarily associated with the crystalline protein inclusion. Indeed, numerous insecticidal proteins active against larvae of different insect orders have been identified within the crystalline inclusions, and some of these proteins have been successfully incorporated in insecticidal formulations and the construction of insect‐pest‐resistant transgenic plants [1, 2, 3].

Insecticidal proteins are structurally diverse and classified on the basis of structure [4]. For example, Cry proteins, the largest family of insecticidal proteins, share a similar three‐domain architecture (domains I, II, and III). Cry proteins are generally considered α‐pore‐forming toxins (α‐PFTs) in which the α‐helical hairpins of domain I form pores in the target cell membrane. A variety of approaches have been employed to observe the formation of pores by many Cry proteins [3]. We previously described pore formation by the mosquito‐larvicidal protein Cry4Aa using artificial lipid bilayers [5]. The group of insecticidal proteins contained in crystalline inclusions also includes a family of α‐PFT‐like proteins, the App proteins [4]. Crystalline inclusions also contain β‐PFTs, which form pores by inserting β‐hairpins into the target cell membrane, as well as β‐PFT‐like proteins. These toxins are classified into several families based on structure and include Cyt, Tpp, Mpp, and Gpp proteins [4]. In particular, the formation of pores in artificial lipid bilayers has been reported for Mpp46Ab [6].

As insecticidal proteins predominantly function as PFTs, pore formation is considered central to their insecticidal activity. Indeed, a correlation between the ion conductance of toxin channel pores and insecticidal activity has been reported for several insecticidal proteins [7, 8, 9, 10]. In addition, an apparent correlation between the cation selectivity of the toxin pores and their insecticidal activity was reported for Mpp46Ab [11, 12] and Cry4Aa [10]. These observations suggest that the ion permeability of toxin pores is closely related to insecticidal activity and that altering the ion permeability of toxin pores could be an effective strategy for enhancing the insecticidal activity of PFTs. In general, electrophysiological analysis using artificial lipid bilayers is a useful approach for studying the formation of pores by insecticidal proteins, especially examining the ion permeability of toxin pores. Many insecticidal proteins reportedly form pores in artificial lipid bilayers [5, 6, 13, 14, 15, 16, 17, 18].

In our previous analyses of Mpp46Ab and Cry4Aa pore formation, we have used several different methods to prepare lipid bilayers. For example, the pore formation of Mpp46Ab was analyzed using lipid bilayers prepared by painting an asolectin (phospholipids from soybean) solution across a small hole (approximately φ200 μm), and the single‐channel conductance was determined to be 103.3 ± 4.1 pS in 150 mm KCl [6]. The ion selectivity of the Mpp46Ab pores was further analyzed using lipid bilayers prepared by liposome fusion [11, 12]. In the case of Cry4Aa pore formation, a solvent‐free lipid bilayer was formed at the tip of the micropipette according to the Tip‐Dip method [19], and the single‐channel conductance was determined to be 187 ± 10 pS in 150 mm KCl [5]. However, such experiments are time‐consuming and sometimes require special skills and apparatus, especially regarding the construction of lipid bilayers and the subsequent formation of toxin pores. It is desirable to develop a simple method for conducting electrophysiologic measurements in order to accelerate the study of channel pores formed by insecticidal proteins.

In the present study, we evaluated the usefulness of agarose gel‐supported lipid bilayers in electrophysiologic measurements using channel pores formed by two structurally different mosquito‐larvicidal proteins, Mpp46Ab and Cry4Aa. In addition, we reconstituted lipid rafts using cholesterol (Chol) and sphingomyelin (SM) in the lipid bilayers at the gel/water interface and investigated the effect of the microdomain on the efficiency of toxin pore formation.

## Results

### Insecticidal proteins

**Fig. 1:**
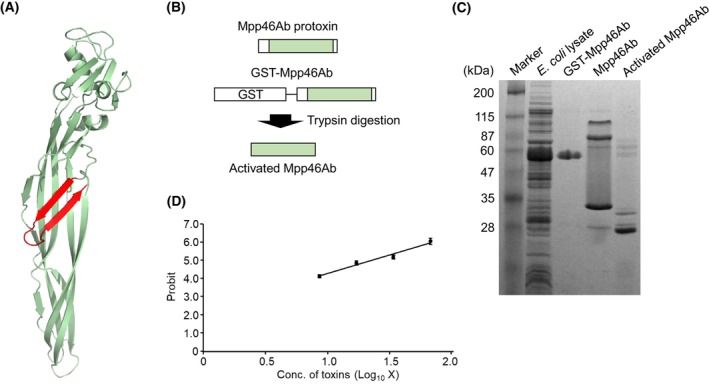
Preparation of recombinant Mpp46Ab toxin. (A) Three‐dimensional structure of Mpp46Ab. The structure model was generated using pymol software [33]. The structure data file was generated using SWISS‐MODEL [34, 35] with the PDB code for the structurally closely related toxin Mpp46Aa (PDB: 2ZTB [36]). The putative transmembrane region (β8‐loop‐β9) is shown in red. (B) Schematic illustration of the activated toxin preparation. (C) SDS/PAGE (10% gel) analysis of Mpp46Abs. A total of 10 μg of protein was used for sonicated *Escherichia coli* cells and 1 μg of purified protein. The experiment was repeated three times with an independently prepared sample, and one representative figure is shown. (D) Mosquito‐larvicidal activity of activated Mpp46Ab. The experiment was repeated three times independently, and the log (dose) vs. probit plot (mean [SD]) is shown.

In the present study, we used two structurally different insecticidal toxins, Mpp46Ab and Cry4Aa. Mpp46Ab is a β‐PFT with an aerolysin‐type architecture (Fig. 1A); the protoxin was expressed as a glutathione *S*‐transferase (GST) fusion and activated using trypsin (Fig. 1B). Sodium dodecyl sulfate–polyacrylamide gel electrophoresis (SDS/PAGE) revealed that the molecular masses of the GST‐Mpp46Ab, Mpp46Ab protoxin, and activated Mpp46Ab were approximately 59, 33, and 28 kDa, respectively, similar to the expected masses (Fig. 1C). Note that GST‐Mpp46Ab is highly purified, but several protein bands of higher molecular mass were observed in the Mpp46Ab protoxin and activated Mpp46Ab (Fig. 1C). Since protein samples of the Mpp46Ab protoxin and activated Mpp46Ab were prepared using purified GST‐Mpp46Ab, those with higher molecular mass were considered to be oligomeric Mpp46Abs generated after cleavage of GST. The formation of oligomeric Mpp46Ab has been frequently observed previously [11, 12]. The activated Mpp46Ab exhibited apparent toxicity against *Culex pipiens* mosquito larvae, with a 50% lethal concentration (LC_50_) (95% confidence interval) of 0.73 (0.65–0.80) μg·mL^−1^ or 25.0 (22.6–27.7) nm (Fig. 1D).

**Fig. 2:**
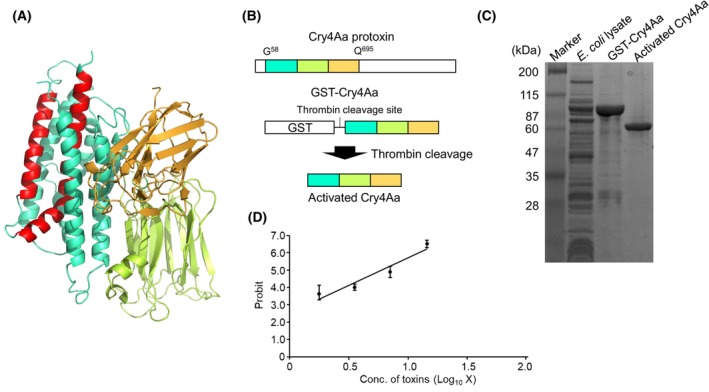
Preparation of recombinant Cry4Aa toxin. (A) Three‐dimensional structure of Cry4Aa activated toxin fragment. The structure model was generated using pymol software with the PDB code for Cry4Aa (PDB: 2C9K [37]). The putative transmembrane region (α4‐loop‐α5) is shown in red. (B) Schematic illustration of the activated toxin preparation. The Cry4Aa active toxin fragment was expressed as a GST fusion and then cleaved using thrombin. C SDS/PAGE (10% gel) analysis of Cry4Aas. A total of 10 μg of protein was used for sonicated *Escherichia coli* cells and 1 μg of purified protein. The experiment was repeated three times with an independently prepared sample, and one representative figure is shown. (D) Mosquito‐larvicidal activity of activated Cry4Aa. The experiment was repeated three times independently, and log (dose) vs. probit plot (mean [SD]) is shown.

By comparison, Cry4Aa is an α‐PFT with a typical three‐domain architecture (Fig. 2A). The Cry4Aa activated toxin fragment (G^58^–Q^695^) expressed as a GST fusion was purified using glutathione beads and then cleaved using thrombin (Fig. 2B). SDS/PAGE revealed that the molecular masses of GST‐Cry4Aa and activated Cry4Aa were approximately 100 and 70 kDa, respectively, similar to the expected masses (Fig. 2C). The 70‐kDa Cry4Aa active toxin fragment exhibited significant toxicity against *C. pipiens* mosquito larvae, with an LC_50_ (95% confidence interval) of 0.42 (0.39–0.45) μg·mL^−1^ or 6.13 (5.71–6.59) nm (Fig. 2D). This was similar to the previously reported LC_50_ (7.70 nm) of the 70‐kDa active Cry4Aa toxin [5]. In this manner, we successfully prepared active toxin fragments of Mpp46Ab and Cry4Aa that exhibited potent mosquito‐larvicidal activity and used these fragments in subsequent electrophysiologic measurements.

### Analysis of Mpp46Ab pores

**Fig. 3:**
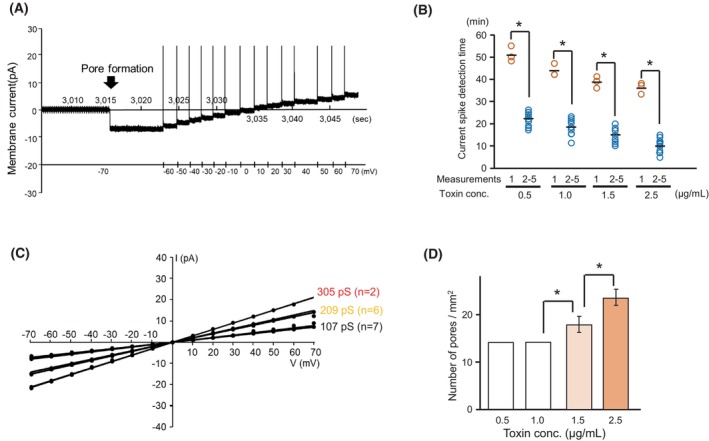
Electrophysiologic analysis of channel pores formed by Mpp46Ab. The measurement was repeated sequentially a total of 5 times using the same apparatus and toxins, and each series of experiments was repeated independently three times. (A) Representative segment of a typical current trace. Arrow shows the current spike indicating pore formation. (B) Time required to detect the current spike indicating pore formation. (C) Representative current–voltage relationship of Mpp46Ab channel pores (toxin concentration 2.5 μg·mL^−1^). (D) Average number of toxin pores detected in 5 sequential measurements, shown as mean (SD). Statistical significance was evaluated using Student's *t* test. Asterisk indicates statistical significance (*P* < 0.05).

The ion permeability of Mpp46Ab pores was analyzed using a symmetric buffer (150 mm KCl, 10 mm Tris/HCl [pH 8.0] in both the *cis* and *trans* chambers). For these measurements, the agarose gel‐supported lipid bilayers were prepared using a lipid solution containing only phosphatidylcholine (PC), and the subsequent formation of Mpp46Ab pores was facilitated by applying voltage (−70 mV). In the measurement, a sudden current spike was observed after placing the *cis* chamber at the interface between the lipid solution and the recording solution in the *trans* chamber (Fig. 3A). This spike was thought to be indicative of Mpp46Ab pore formation, as no similar current transition was observed in the recording lacking Mpp46Ab toxin. The current appeared to indicate the pores remained in a stable open state for at least several minutes, similar to measurements using our previous conventional method [11, 12]. These data thus demonstrated that the agarose gel‐supported lipid bilayers prepared in this study are suitable for the measurement of the ion permeability of Mpp46Ab pores. In this study, measurements were continually repeated a total of 5 times using the same apparatus and toxins, but only the first measurement required a long period of time before the current spike was detected (Fig. 3B). This result suggests that the reconstitution of the lipid monolayer at the interface takes some time after the lipid solution is placed over the recording solution in the *trans* chamber. Alternatively, pore formation by Mpp46Ab is initiated, at least in part, immediately after the lipid solution is placed over the recording solution, and the time required for the second and subsequent measurements may be shorter. In addition, the elapsed time before the current spike was detected seemed to be shorter at higher Mpp46Ab concentrations, but the difference was not statistically significant within the range of toxin concentrations examined in this study (Fig. 3B).

Following detection of the current spike, the current was recorded between −70 and +70 mV and plotted against the corresponding applied voltage to generate current–voltage relationships (Fig. 3C). The measurement was continually repeated a total of 5 times using the same apparatus and toxins, and a series of measurements were carried out 3 times using independently prepared Mpp46Ab samples. The current–voltage relationship was generally linear, but lines exhibiting multiple conductance levels were observed, especially in measurements at higher toxin concentrations (> 1.5 μg·mL^−1^). Furthermore, although the same apparatus and toxin were used for each repeated measurement, different current levels were detected in each of the five sequential measurements. Notably, the observed conductance was almost universally in multiples of 100 pS; therefore, conductance values > 100 pS were attributed to the presence of multiple toxin pores (Fig. 3C). Indeed, no lines indicating a conductance value > 100 pS were observed in measurements conducted at lower toxin concentrations (< 1.0 μg·mL^−1^). The single‐channel conductance of the Mpp46Ab pores was therefore determined to be 104.3 ± 3.9 pS (*n* = 60) in symmetric buffer containing 150 mm KCl, similar to the value (103.3 ± 4.1 pS) previously determined using our conventional method [6].

It should be noted that the average number of Mpp46Ab pores detected in the five sequential measurements was similar between measurements performed with different Mpp46Ab preparations, and the values appeared to correlate with toxin concentration (Fig. 3D). This indicates that this value may be a good indicator of toxin pore formation efficiency.

### Analysis of Cry4Aa pores

**Fig. 4:**
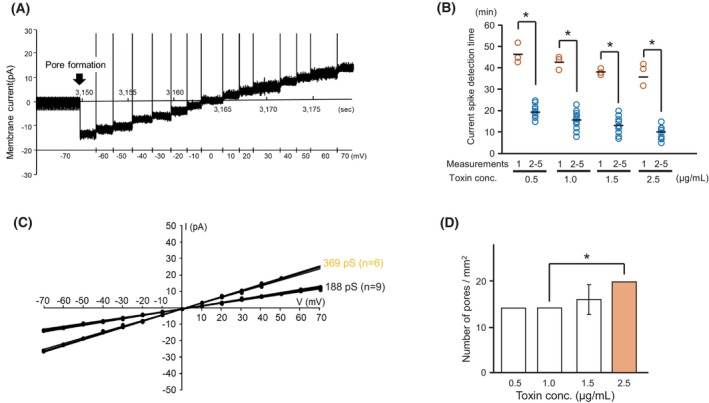
Electrophysiologic analysis of channel pores formed by Cry4Aa. The measurement was repeated sequentially a total of 5 times using the same apparatus and toxins, and each series of experiments was repeated independently 3 times. (A) Representative segment of a typical current trace. Arrow shows the current spike indicating pore formation. (B) Time required to detect the current spike indicating pore formation. (C) Representative current–voltage relationship of Cry4Aa channel pores (toxin concentration 2.5 μg·mL^−1^). (D) Average number of toxin pores detected in 5 sequential measurements, shown as mean (SD). Statistical significance was evaluated using Student's *t* test. Asterisk indicates statistical significance (*P* < 0.05).

The ion permeability of Cry4Aa pores was analyzed in a manner similar to that of Mpp46Ab pores described above. Cry4Aa pores exhibited a profile very similar to that of Mpp46Ab pores, except with regard to the single‐channel conductance value. Briefly, a sudden current spike indicating pore formation was also observed in the Cry4Aa measurements (Fig. 4A). The current appeared to indicate the pores remained in a stable open state for at least several minutes, similar to measurements using our previous conventional method [5]. The time lag before detection of the current spike was very similar to that observed in Mpp46Ab pore formation, with a significantly longer time observed only for the first measurement and an apparent correlation with Cry4Aa concentration (Fig. 4B). The current–voltage relationship of the Cry4Aa pores was linear, but two conductance levels were observed (Fig. 4C), especially for measurements conducted at higher toxin concentrations (> 2.0 μg·mL^−1^). As the higher conductance (369 pS) was almost twice the value of the lower conductance (188 pS), the higher conductance current was thought to result from the formation of two toxin pores (Fig. 4C). The single‐channel conductance of the Cry4Aa pores was 187.5 ± 5.9 pS (*n* = 59) in symmetric buffer containing 150 mm KCl, similar to the value (187 ± 10 pS) previously determined using the conventional method [5]. Similar to the results of Mpp46Ab measurements, the average number of Cry4Aa pores was relatively similar in each of the five continual measurements, and the values seemed to correlate with toxin concentration (Fig. 4D).

### The bilayer lipid composition affects the efficiency of toxin pore formation

We also investigated the effect of the lipid bilayer composition on toxin pore formation. Different types of lipid bilayers were constructed using four different lipid solutions (*n*‐decane) containing PC, Chol, and/or SM at different ratios (PC : Chol : SM = 9 : 1 : 0, 9 : 0 : 1, 8 : 1 : 1, and 6 : 2 : 2) in addition to a lipid solution of PC only (10 : 0 : 0).

**Fig. 5:**
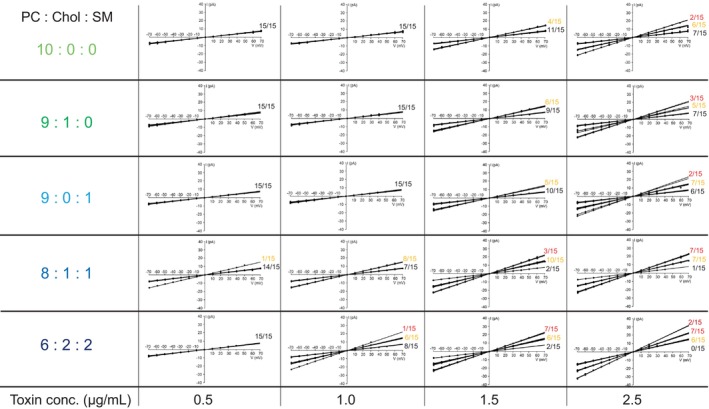
Current–voltage relationship for Mpp46Ab channel pores measured using different lipid bilayers. Channel currents recorded in symmetric 150 mm KCl solutions were plotted against applied voltage. Measurements were repeated sequentially a total of 5 times using the same apparatus and toxins, and each series of experiments was repeated independently 3 times. Lipid bilayers were constructed using different lipid cocktails (in *n*‐decane). Chol, cholesterol; PC, phosphatidylcholine; SM, sphingomyelin.

**Fig. 6:**
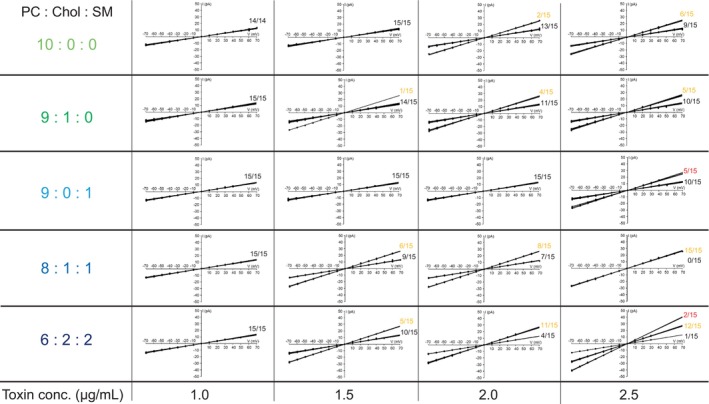
Current–voltage relationship for Cry4Aa channel pores measured using different lipid bilayers. Channel currents recorded in symmetric 150 mm KCl solutions were plotted against applied voltage. Measurements were repeated sequentially a total of 5 times using the same apparatus and toxins, and each series of experiments was repeated independently 3 times. Lipid bilayers were constructed using different lipid cocktails (in *n*‐decane). Chol, cholesterol; PC, phosphatidylcholine; SM, sphingomyelin.

Interestingly, the Mpp46Ab pores exhibited very similar channel currents in all measurements using the different lipid bilayer types (data not shown). The current was recorded between −70 and +70 mV and plotted against the corresponding applied voltage to generate current–voltage relationships (Fig. 5). The single‐channel conductance values of Mpp46Ab pores determined in lipid bilayers consisting of PC : Chol : SM at 9 : 1 : 0, 9 : 0 : 1, 8 : 1 : 1, and 6 : 2 : 2 were 106.7 ± 5.3 (*n* = 60), 107.2 ± 15.3 (*n* = 60), 109.2 ± 2.5 (*n* = 60), and 111.2 ± 14.2 pS (*n* = 60), respectively. These values were similar to the value (104.3 ± 3.9 pS) determined for the lipid bilayer containing only PC. Similarly, channel currents were not altered in any of the Cry4Aa pore measurements (Fig. 6). The single‐channel conductance values of Cry4Aa pores determined in lipid bilayers consisting of PC : Chol : SM at 9 : 1 : 0, 9 : 0 : 1, 8 : 1 : 1, and 6 : 2 : 2 were 190.4 ± 9.4 (*n* = 60), 187.2 ± 7.0 (*n* = 60), 191.2 ± 3.4 (*n* = 60), and 192.5 ± 4.5 pS (*n* = 60), respectively. These values were similar to the value (187.5 ± 5.9 pS) determined using lipid bilayers containing only PC. These results suggest that lipid bilayer composition has little or no effect on Mpp46Ab and Cry4Aa pore structure.

**Fig. 7:**
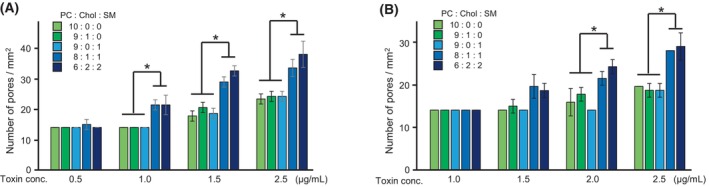
Effect of lipid composition on toxin pore formation. Average number (mean [SD]) of toxin pores detected in the five sequential measurements is shown. Statistical significance was evaluated using Student's *t* test. Asterisk indicates statistical significance (*P* < 0.05). (A) Mpp46Ab; (B) Cry4Aa. Lipid bilayers were constructed using different lipid cocktails (in *n*‐decane). Chol, cholesterol; PC, phosphatidylcholine; SM, sphingomyelin.

On the other hand, the average number of Mpp46Ab pores detected in the 5 sequential measurements was significantly higher in lipid bilayers consisting of PC : Chol : SM at 8 : 1 : 1 and 6 : 2 : 2 compared with the number of pores in lipid bilayers consisting of PC : Chol : SM at 10 : 0 : 0, 9 : 1 : 0, and 9 : 0 : 1, particularly in measurements conducted at toxin concentrations > 1.0 μg·mL^−1^ (Fig. 7A). A similar increase in the number of pores was also observed in Cry4Aa measurements, especially at toxin concentrations > 2.0 μg·mL^−1^ (Fig. 7B). These data thus demonstrate that the addition of both Chol and SM to the lipid bilayers facilitates the formation of pores by both Mpp46Ab and Cry4Aa.

## Discussion

**Fig. 8:**
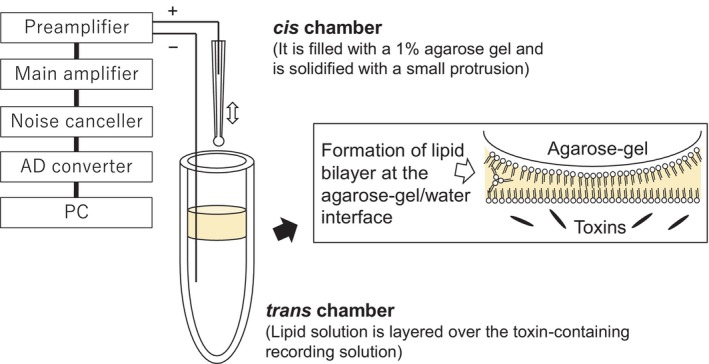
Schematic representation of the apparatus and procedure for bilayer formation at the agarose gel/water interface. The *cis* chamber was inserted into a lipid solution in the *trans* chamber, and the protrusion of 1% agarose gel was placed at the interface between the lipid solution and the recording solution to produce a lipid bilayer.

In this study, we used a simple experimental apparatus consisting of a generic gel‐loading tip filled with an agarose gel (*cis* chamber) and a generic 0.6‐mL microtube (*trans* chamber) to prepare the agarose gel‐supported lipid bilayers (Fig. 8). Two structurally different mosquito‐larvicidal toxins, Mpp46Ab and Cry4Aa, were examined, and the single‐channel conductance values of the toxin‐produced pores determined in this study were very similar to the values previously determined using our conventional method. This consistency suggests that the agarose gel‐supported lipid bilayers are useful for measuring channel currents through different types of toxin pores. A remarkable feature of this system is that the lipid bilayers can be easily reconstructed even after the lipid bilayer has been disrupted. Indeed, ion permeability measurements of the toxin pores could be repeated sequentially at least five times using the same apparatus and toxins. Presumably, the measurement can be repeated many more than five times. Interestingly, the time required to detect the channel current was typically 30–60 min for the first measurement, similar to results obtained using our conventional method [5, 6]. However, the second and subsequent measurements required significantly less time (5–30 min, Figs 3B and 4B). The ease of bilayer reconstruction observed in this study may be of great advantage in studies conducting electrophysiologic measurements of insecticidal toxins. In addition, the average number of pores detected over the five sequential measurements was similar between measurements performed with different toxin preparations, and the values appeared to correlate with toxin concentration (Figs 3D and 4D). Therefore, we considered this value to be a good indicator of the efficiency of toxin pore formation. In addition to the single‐channel analysis described above, this system is also expected to be useful in situations where an asymmetric buffer containing different salts is used. The ion selectivity of the toxin pores, particularly the anion–cation selectivity, affects the insecticidal activity of the Mpp46Ab and Cry4Aa toxins, and the selectivity can be altered by mutagenesis targeting the transmembrane region. The agarose gel‐supported lipid bilayer is shown to be a valuable tool for measuring channel currents through toxin pores and will contribute to the efficient selection of improved mutant toxins.

The present study also investigated the effects of the lipid bilayer components on toxin pore formation. Various lipid bilayers were constructed using lipid solutions containing PC, Chol, and SM in varying proportions. Both Chol and SM are major components of lipid rafts, which are microdomains in the eukaryotic cell membrane [20]. It is widely accepted that lipid rafts not only regulate various cellular metabolic processes and responses to extracellular stimuli but also affect a wide range of physiologic and pathologic processes [21]. In these analyses, very similar single‐channel conductance values were determined in all measurements, despite differences in lipid bilayer composition (PC : Chol : SM = 10 : 0 : 0, 9 : 1 : 0, 9 : 0 : 1, 8 : 1 : 1, and 6 : 2 : 2), suggesting that the ion permeability through the toxin pores is not affected by the lipid bilayer composition. By contrast, the average number of detected toxin pores was significantly higher in lipid bilayers composed of PC : Chol : SM at 8 : 1 : 1 and 6 : 2 : 2 compared with the number in lipid bilayers composed of PC : Chol : SM at 10 : 0 : 0, 9 : 1 : 0, and 9 : 0 : 1 (Figs 5, 6, 7). This difference suggests that the incorporation of both Chol and SM in the lipid bilayers facilitates pore formation by both Mpp46Ab and Cry4Aa. In particular, the presence of lipid rafts was shown to be essential for the toxicity of many bacterial pore‐forming toxins. Lysenin, a pore‐forming toxin from earthworms, binds SM in lipid rafts [22]. Similarly, the activity of cholera toxin requires both Chol and sphingolipids in lipid rafts [23]. Listeriolysin O, a pore‐forming toxin from the facultative intracellular pathogen *Listeria*, binds to Chol‐containing membranes and oligomerizes to form pores [24]. Aerolysin functions via a glycosylphosphatidylinositol (GPI)‐anchored protein present in lipid rafts [25]. With respect to *B. thuringiensis* insecticidal proteins, lipid rafts are thought to act primarily as platforms for the recruitment of toxin receptors such as GPI‐anchored proteins. For example, Cry4Ba is an insecticidal protein from *B. thuringiensis* subsp. *israelensis*, and structurally, Cry4Ba is closely related to Cry4Aa used in the present study. When incubated with brush border membrane vesicles extracted from *Aedes aegypti* mosquito larvae, Cry4Ba localizes to lipid rafts with GPI‐anchored proteins [26]. Similarly, several putative receptors for lepidopteran‐specific Cry1A toxins, such as the 120‐ and 170‐kDa aminopeptidases from *Heliothis virescens* and the 120‐kDa aminopeptidase from *Manduca sexta*, partition preferentially into lipid rafts, and the presence and proper integrity of lipid rafts have been proposed as a prerequisite for Cry1A pore formation and toxicity [27]. In addition, Mpp46Aa (designated parasporin‐2Aa), which is structurally closely related to Mpp46Ab used in the present study, also oligomerizes and forms pores in lipid rafts [28]. Considering these and our current results, in addition to recruiting toxin receptors such as GPI‐anchored proteins, raft microdomains present in the lipid bilayer appear to play a key role in membrane insertion and pore formation by *B. thuringiensis* insecticidal proteins. Thus, this system may be a valuable tool for determining the specific lipids that are important for toxin pore formation.

## Materials and methods

### Preparation of recombinant insecticidal proteins

The expression vector pGST‐Cry46Ab‐S1 [6] was used to produce recombinant Mpp46Ab, with the Mpp46Ab protoxin expressed as a GST fusion. GST‐Mpp46Ab was purified using glutathione‐Sepharose 4B (GE Healthcare Bio‐Sciences, AB, Uppsala, Sweden) according to the manufacturer's instructions. Purified GST‐Mpp46Ab was then activated using a trypsin‐immobilized column prepared as previously described [6]. Similarly, to produce recombinant Cry4Aa, we used the expression vector pGST‐Cry4Aa‐S1 [29] to express the Cry4Aa active toxin fragment (G^58^–Q^695^) as a GST fusion. The Cry4Aa active toxin fragment was purified using glutathione‐Sepharose 4B (GE Healthcare) and then cleaved from the resin using thrombin (Cytiva, Tokyo, Japan), as previously described [5].

Protein concentration was estimated using a protein assay dye reagent (Bio‐Rad Laboratories, Inc., Hercules, CA, USA) with bovine serum albumin as the standard. Proteins were analyzed by SDS/PAGE, followed by visualization of protein bands by staining the gel with Coomassie brilliant blue (CBB stain one; Nacalai Tesque, Inc., Kyoto, Japan). The biological activity of activated Mpp46Ab and Cry4Aa was analyzed using a bioassay with *C. pipiens* mosquito larvae as described previously [12]. Mosquito larvae were reared from eggs kindly provided by the Research and Development Laboratory, Dainihon Jochugiku Co., Ltd. (Osaka, Japan). Larval mortality was recorded 48 h after toxin administration, and the LC_50_ value with a 95% confidence interval was determined using PROBIT analysis [30]. Bioassays were repeated three times using independently prepared samples.

### Experimental apparatus for electrophysiologic analysis

The method used to construct artificial lipid bilayers at the hydrogel/water interface was previously developed for the electrophysiologic analysis of ion channels [31]. The method enables the incorporation of different types of ion channel proteins into bilayers within a short time. In the present study, we utilized agarose gel‐supported lipid bilayers in order to facilitate analyses of pores formed by insecticidal toxins.

**Fig. 9:**
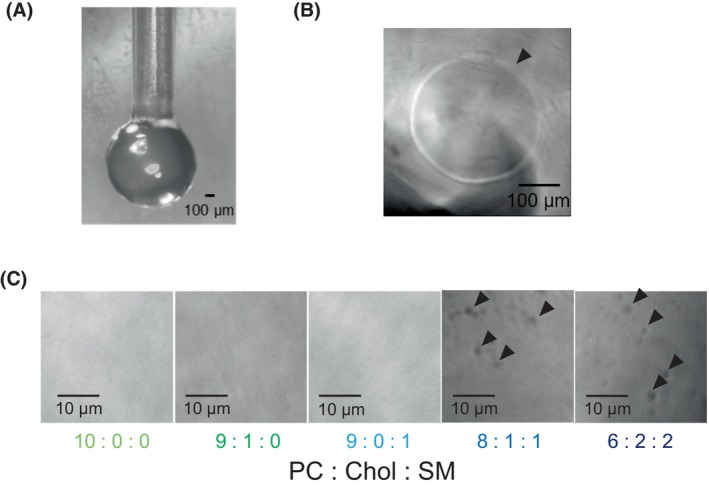
Lipid bilayers formed at the agarose gel/water interface. (A) The agarose gel protruding from the tip in a spherical shape. Bar, 100 μm. (B) Microscopic observation of the interface between the lipid solution and the recording solution. The boundary (arrowhead) between the bilayer and the bulky layer was clearly visible. Bar, 100 μm. (C) Microscopic observation of the lipid bilayer area. Bar, 10 μm. Many small dark spots (arrowhead) were observed in the membranes prepared with PC, Chol, and SM. The experiment was repeated five times, and one representative figure is shown. Chol, cholesterol; PC, phosphatidylcholine; SM, sphingomyelin.

The agarose gel‐supported lipid bilayers were constructed according to a previously reported procedure [31], with some modifications. Briefly, the experimental apparatus consisted of two chambers (*cis* and *trans*), such that the voltage in the *cis* chamber was connected to a patch‐clamp amplifier by a Ag/AgCl electrode‐defined membrane potential (Fig. 8). A generic gel‐loading tip was used as the *cis* chamber. Using a generic micropipette, the tip was filled with a solution of 1% agarose dissolved in recording solution (150 mm KCl and 10 mm Tris/HCl [pH 8.0]) by heating and solidified with a small protrusion (approximately 1 μL) from the tip. The agarose gel protruding from the tip assumed a spherical shape with a diameter of approximately 1 mm (Fig. 9A). A generic 0.6‐mL microtube was used as the *trans* chamber. Recording solution (300 μL) containing activated toxin polypeptides was poured into the microtube, and 100 μL of a lipid solution was layered over the recording solution layer. In this study, L‐α‐PC (PC from egg; Nacalai Tesque), Chol (Nacalai Tesque), and SM from egg (Avanti Polar Lipids, Inc., Alabaster, AL, USA) were independently dissolved in *n*‐decane at a concentration of 40 mg·mL^−1^ by sonication and then mixed in different ratios to prepare lipid solutions.

### Measurement procedure

All manipulations were conducted at room temperature or 25 °C. To produce lipid bilayers, the *cis* chamber was inserted into a lipid solution in the *trans* chamber, and the protrusion of 1% agarose gel was placed at the interface between the lipid solution and recording solution (Fig. 8). When the interface was observed by light microscopy (Axio Observer A1; Carl Zeiss, Göttingen, Germany) from the recording solution side, the formation of a ring indicating the boundary between the lipid bilayer and the bulky layer had usually occurred within 5 min (Fig. 9B). The size of the ring was about 0.3 mm in diameter and did not change significantly with the type of lipid solutions used. Interestingly, many small dark spots were observed in the membranes prepared with PC, Chol, and SM, but not in the membrane lacking at least one of Chol and SM (Fig. 9C). We considered that these spots to be regions of different thickness and refractive index from the rest of the bilayer, caused by differences in Chol and SM content and lipid packing. Using a lipid solution containing PC, Chol, and SM, the formation of lipid rafts in phase‐separated droplet interface bilayers has been previously observed by fluorescence microscopy and interferometric scattering microscopy [32]. This suggests that the lipid raft microdomains can be formed in the lipid bilayers constructed by our system. Thus, its efficiency is unclear, we thought that Chol and SM are incorporated into the PC membrane and formed lipid raft‐like microdomains.

In this study, if a typical current spike indicative of toxin pore formation in lipid bilayers was detected within 5 min of measurement initiation, the current was recorded between −70 and +70 mV. If no such spike was detected, the *cis* chamber was moved upward and then reinserted. In the present study, after completion of the first measurement, the measurement was repeated sequentially a total of 5 times using the same apparatus and toxins. If the typical current spike was not detected within 2.5 h, the measurement was stopped, and the *cis* chamber was re‐prepared. However, such a failure was very rare, occurring only once when measuring a low concentration (1 μg·mL^−1^) of Cry4Aa (Fig. 6). A series of experiments was repeated 3 times using independently prepared insecticidal proteins.

Data were analyzed using pclamp software, ver. 11.1 (Axon Instruments, Roster City, CA, USA). In each case, current was plotted against the corresponding applied voltage to generate current–voltage relationship graphs. Channel conductance was determined from the slope of the current–voltage relationship. Statistical significance was evaluated using Student's *t* test.

## Conflict of interest

The authors declare no conflict of interest.

## Author contributions

TO and TH performed the biological experiments. TO performed the electrophysiologic analyses with assistance from TT, MA, MH, and TI. TO and TH analyzed the data from all experiments. TH wrote the manuscript, with contributions from MH and TI. TH, TI, and MH supervised the project. TH acquired funding for the research.

## Peer review

The peer review history for this article is available at https://www.webofscience.com/api/gateway/wos/peer‐review/10.1111/febs.70070.

## Data Availability

Data are available from the authors upon reasonable request.
